# Tetra­aqua­bis[3-(3-pyrid­yl)-5-(4-pyrid­yl)-1,2,4-triazolido]nickel(II) dihydrate

**DOI:** 10.1107/S1600536809047102

**Published:** 2009-11-14

**Authors:** Yun-Liang Zhang, Ti-Lou Liu, Shuang-Jiao Sun

**Affiliations:** a Department of Pharmacy, Shaoyang Medical College, Shaoyang, Hunan 422000, People’s Republic of China

## Abstract

In the title compound, [Ni(C_12_H_8_N_5_)_2_(H_2_O)_4_]·2H_2_O, the Ni^II^ atom is coordinated by the two N atoms [Ni—N = 2.094 (3) Å] and four O atoms [Ni—O = 2.063 (3)–2.083 (2) Å] in a distorted octa­hedral geometry. The mol­ecule is centrosymmetric and the Ni^II^ atom is located on an inversion center. Inter­molecular O—H⋯N and O—H⋯O hydrogen bonds link the complex into a three-dimensional supra­molecular framework.

## Related literature

For hydrogen-bond inter­actions in biological systems, see: Deisenhofer & Michel (1989 ▶). For supra­molecular assembly through hydrogen bonds, see: Beatty (2003 ▶); Li *et al.* (2006 ▶); Russell & Ward (1996 ▶). For related structures, see: Liu *et al.* (2008 ▶); Liu & Zhang (2009 ▶); Rarig *et al.* (2001 ▶). 
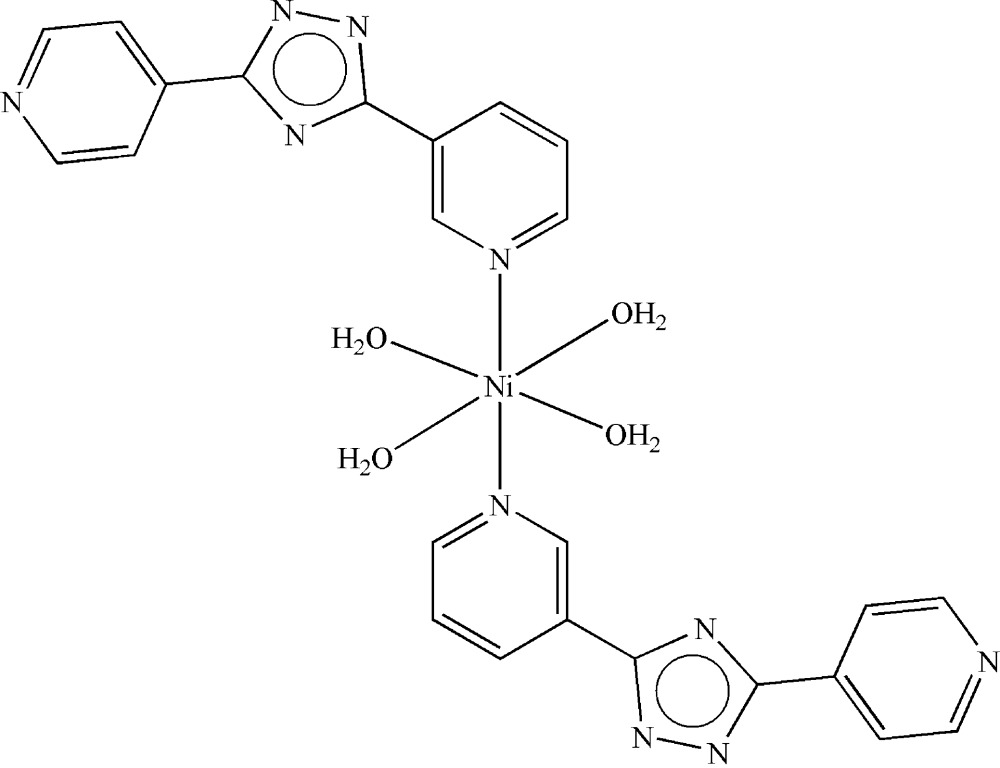


## Experimental

### 

#### Crystal data

[Ni(C_12_H_8_N_5_)_2_(H_2_O)_4_]·2H_2_O
*M* *_r_* = 611.25
Triclinic, 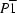

*a* = 8.2240 (16) Å
*b* = 9.1990 (18) Å
*c* = 9.3850 (19) Å
α = 90.70 (3)°
β = 104.96 (3)°
γ = 96.47 (3)°
*V* = 680.9 (2) Å^3^
*Z* = 1
Mo *K*α radiation
μ = 0.77 mm^−1^
*T* = 293 K
0.20 × 0.12 × 0.08 mm

#### Data collection

Bruker SMART CCD area-detector diffractometer
Absorption correction: multi-scan (*SADABS*; Bruker, 1998 ▶)’ *T*_min_ = 0.866, *T*_max_ = 0.943
4042 measured reflections
2437 independent reflections
2258 reflections with *I* > 2σ(*I*)
*R*_int_ = 0.040

#### Refinement

*R*[*F* ^2^ > 2σ(*F* ^2^)] = 0.052
*wR*(*F* ^2^) = 0.150
*S* = 0.99
2437 reflections
211 parameters
9 restraints
H atoms treated by a mixture of independent and constrained refinement
Δρ_max_ = 0.55 e Å^−3^
Δρ_min_ = −0.85 e Å^−3^

### 

Data collection: *SMART* (Bruker, 1998 ▶); cell refinement: *SAINT* (Bruker, 1998 ▶); data reduction: *SAINT*; program(s) used to solve structure: *SHELXS97* (Sheldrick, 2008 ▶); program(s) used to refine structure: *SHELXL97* (Sheldrick, 2008 ▶); molecular graphics: *SHELXTL* (Sheldrick, 2008 ▶); software used to prepare material for publication: *SHELXTL*.

## Supplementary Material

Crystal structure: contains datablocks global, I. DOI: 10.1107/S1600536809047102/bg2306sup1.cif

Structure factors: contains datablocks I. DOI: 10.1107/S1600536809047102/bg2306Isup2.hkl

Additional supplementary materials:  crystallographic information; 3D view; checkCIF report

## Figures and Tables

**Table 1:** Hydrogen-bond geometry (Å, °)

*D*—H⋯*A*	*D*—H	H⋯*A*	*D*⋯*A*	*D*—H⋯*A*
O1—H1*A*⋯N5^i^	0.83 (2)	1.92 (3)	2.751 (4)	179 (3)
O1—H1*B*⋯N3^ii^	0.83 (3)	1.95 (3)	2.750 (4)	162 (3)
O2—H2*A*⋯O3^iii^	0.83 (3)	1.93 (3)	2.751 (4)	171 (3)
O2—H2*B*⋯N4^iv^	0.84 (3)	1.96 (3)	2.791 (4)	169 (3)
O3—H3*A*⋯N2^v^	0.82 (5)	2.10 (5)	2.911 (4)	170 (3)
O3—H3*B*⋯N4^vi^	0.82 (4)	2.20 (5)	2.944 (4)	151 (3)

Symmetry codes: (i) 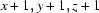; (ii) 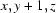; (iii) 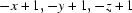; (iv) 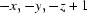; (v) 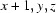; (vi) 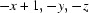.

**Fig. 1.**
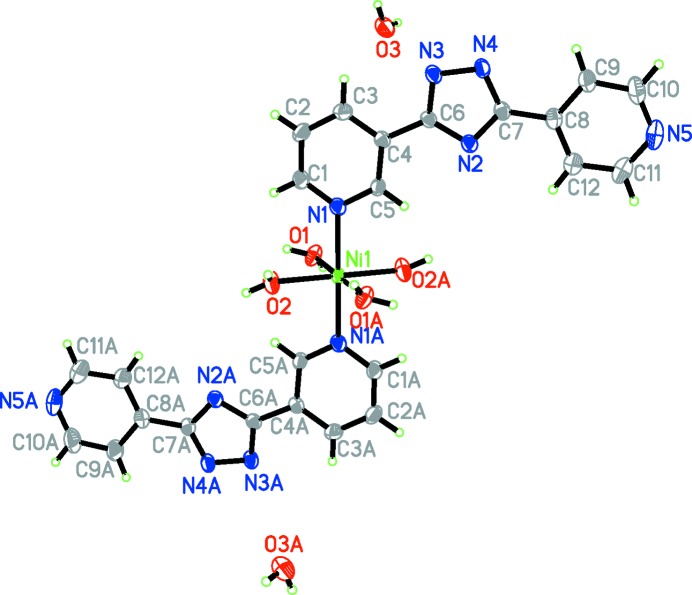
A view of the molecular structure of (I) with the atom-numbering scheme and 50% displacement ellipsoids (arbitrary spheres for the H atoms). Atoms with the suffix A are generated by the symmetry operation (-x, -y + 1, -z + 1).

**Fig. 2.**
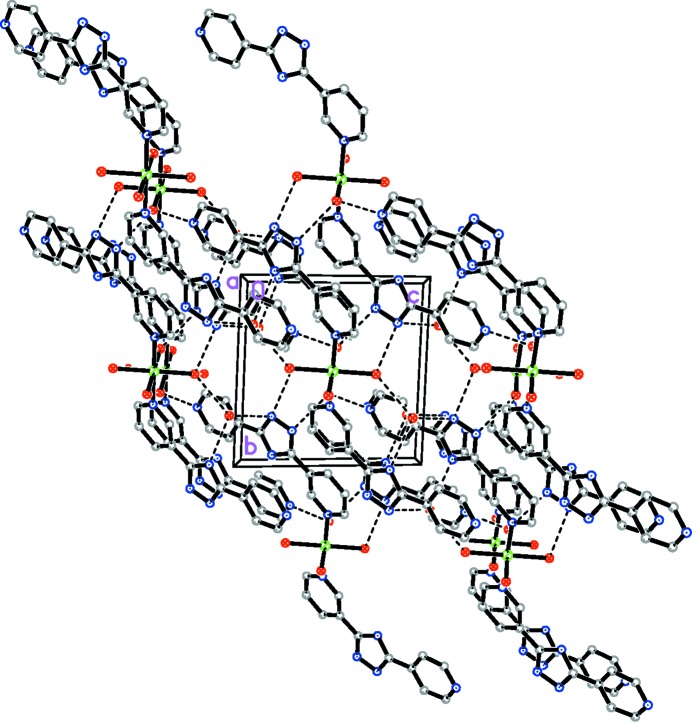
The 3-D network of (I).

## supplementary crystallographic information

### Comment

The hydrogen bond interaction plays a important role in some biological systems (Deisenhofer & Michel, 1989). Supramolecular assembly through hydrogen bonds has been extensively exploited to generate extended one-, two- and three-dimensional structures (Beatty *et al.*, 2003; Li *et al.*, 2006; Russell & Ward, 1996). As part of this ongoing work (Liu *et al.*, 2009), We present here the synthesis and structural characterization of the title nickel complex, [Ni(C_12_H_8_N_5_)_2_(H_2_O)_4_].2H_2_O, (I).

The molecule of the title complex, (Fig. 1), is centrosymmetric, so pairs of equivalent ligands lie *trans* to each other in a slightly distorted octahedral coordination geometry, *cis* angles deviating from 90° by less than 2°. with Ni—O bond length in the range 2.063–2.083 Å and Ni—N bond length 2.094 Å. These bond distances compare well with those in the literature (Liu *et al.*, 2008; Rarig *et al.*, 2001). Molecules are linked by O—H···O and O—H···N hydrogen bonds (Fig. 2, Table 1).

### Experimental

Ni(NO_3_)_2_.4H_2_O (0.5 mmol, 0.145 g), 1*H*-3-(3-pyridyl)-5-(4-pyridyl)-1,2,4-triazole (0.5 mmol, 0.112 g), and water (12 ml) were placed in a 23-ml Teflon-lined Parr bomb. The bomb was heated at 453 K for 3 d. The green block-shapped crystals were filtered off and washed with water and acetone (yield 33%, based on Ni).

### Refinement

Hydrogen atoms of water molecules were located in a difference Fourier map and refined with distance restraints of O—H = 0.82 (2) Å and H···H = 1.35 (2) Å. H atoms on C atom were positoned geometrically and refined using a riding model, with C—H = 0.93 Å, in all cases with U(H)= 1.2/1.5× U_eqiv_(Host)

### Crystal data

**Table:**

[Ni(C_12_H_8_N_5_)_2_(H_2_O)_4_]·2H_2_O	*Z* = 1
*M**_r_* = 611.25	*F*(000) = 318
Triclinic, *P*1	*D*_x_ = 1.491 Mg m^−^^3^
Hall symbol: -P 1	Mo *K*α radiation, λ = 0.71073 Å
*a* = 8.2240 (16) Å	Cell parameters from 2567 reflections
*b* = 9.1990 (18) Å	θ = 1.5–25.3°
*c* = 9.3850 (19) Å	µ = 0.77 mm^−^^1^
α = 90.70 (3)°	*T* = 293 K
β = 104.96 (3)°	Block, green
γ = 96.47 (3)°	0.20 × 0.12 × 0.08 mm
*V* = 680.9 (2) Å^3^	

### Data collection

**Table:**

Bruker SMART CCD area-detector diffractometer	2437 independent reflections
Radiation source: fine-focus sealed tube	2258 reflections with *I* > 2σ(*I*)
graphite	*R*_int_ = 0.040
φ and ω scans	θ_max_ = 25.2°, θ_min_ = 3.1°
Absorption correction: multi-scan (*SADABS*; Bruker, 1998)'	*h* = −9→9
*T*_min_ = 0.866, *T*_max_ = 0.943	*k* = −9→11
4042 measured reflections	*l* = −11→11

### Refinement

**Table:**

Refinement on *F*^2^	Primary atom site location: structure-invariant direct methods
Least-squares matrix: full	Secondary atom site location: difference Fourier map
*R*[*F*^2^ > 2σ(*F*^2^)] = 0.052	Hydrogen site location: inferred from neighbouring sites
*wR*(*F*^2^) = 0.150	H atoms treated by a mixture of independent and constrained refinement
*S* = 0.99	*w* = 1/[σ^2^(*F*_o_^2^) + (0.0912*P*)^2^ + 0.8*P*] where *P* = (*F*_o_^2^ + 2*F*_c_^2^)/3
2437 reflections	(Δ/σ)_max_ < 0.001
211 parameters	Δρ_max_ = 0.55 e Å^−^^3^
9 restraints	Δρ_min_ = −0.85 e Å^−^^3^

### Special details

**Table:**

Geometry. All e.s.d.'s (except the e.s.d. in the dihedral angle between two l.s. planes) are estimated using the full covariance matrix. The cell e.s.d.'s are taken into account individually in the estimation of e.s.d.'s in distances, angles and torsion angles; correlations between e.s.d.'s in cell parameters are only used when they are defined by crystal symmetry. An approximate (isotropic) treatment of cell e.s.d.'s is used for estimating e.s.d.'s involving l.s. planes.
Refinement. Refinement of *F*^2^ against ALL reflections. The weighted *R*-factor *wR* and goodness of fit *S* are based on *F*^2^, conventional *R*-factors *R* are based on *F*, with *F* set to zero for negative *F*^2^. The threshold expression of *F*^2^ > σ(*F*^2^) is used only for calculating *R*-factors(gt) *etc*. and is not relevant to the choice of reflections for refinement. *R*-factors based on *F*^2^ are statistically about twice as large as those based on *F*, and *R*- factors based on ALL data will be even larger.

### Fractional atomic coordinates and isotropic or equivalent isotropic displacement parameters (Å^2^)

**Table:**

	*x*	*y*	*z*	*U*_iso_*/*U*_eq_	
Ni1	0.0000	0.5000	0.5000	0.0217 (2)	
C1	0.2767 (4)	0.3042 (4)	0.6004 (4)	0.0291 (7)	
H1	0.3256	0.3814	0.6683	0.035*	
C2	0.3637 (5)	0.1847 (4)	0.5989 (4)	0.0347 (8)	
H2	0.4679	0.1806	0.6663	0.042*	
C3	0.2938 (4)	0.0712 (4)	0.4959 (4)	0.0299 (8)	
H3	0.3508	−0.0100	0.4928	0.036*	
C4	0.1385 (4)	0.0799 (3)	0.3976 (3)	0.0211 (6)	
C5	0.0578 (4)	0.2022 (3)	0.4086 (3)	0.0235 (7)	
H5A	−0.0480	0.2078	0.3440	0.028*	
C6	0.0580 (4)	−0.0361 (3)	0.2837 (3)	0.0214 (6)	
C7	−0.1047 (4)	−0.1550 (3)	0.1011 (3)	0.0231 (7)	
C8	−0.2408 (4)	−0.2007 (4)	−0.0305 (4)	0.0270 (7)	
C9	−0.2480 (5)	−0.3324 (4)	−0.1091 (4)	0.0382 (9)	
H9A	−0.1643	−0.3938	−0.0785	0.046*	
C10	−0.3798 (6)	−0.3703 (5)	−0.2319 (5)	0.0480 (11)	
H10A	−0.3804	−0.4570	−0.2841	0.058*	
C11	−0.4989 (5)	−0.1649 (5)	−0.2061 (4)	0.0428 (10)	
H11A	−0.5863	−0.1074	−0.2379	0.051*	
C12	−0.3699 (5)	−0.1149 (4)	−0.0848 (4)	0.0363 (8)	
H12A	−0.3689	−0.0242	−0.0395	0.044*	
N1	0.1255 (4)	0.3132 (3)	0.5083 (3)	0.0253 (6)	
N2	−0.0797 (3)	−0.0233 (3)	0.1726 (3)	0.0243 (6)	
N3	0.1159 (4)	−0.1651 (3)	0.2840 (3)	0.0263 (6)	
N4	0.0088 (4)	−0.2439 (3)	0.1640 (3)	0.0276 (6)	
N5	−0.5067 (4)	−0.2906 (4)	−0.2810 (4)	0.0442 (9)	
O1	0.2181 (3)	0.6258 (3)	0.4839 (3)	0.0321 (6)	
O2	0.0796 (4)	0.5155 (3)	0.7297 (3)	0.0353 (6)	
O3	0.8076 (4)	0.2539 (3)	0.0662 (3)	0.0405 (7)	
H1A	0.302 (4)	0.650 (5)	0.555 (3)	0.059 (15)*	
H2A	0.122 (5)	0.580 (3)	0.796 (4)	0.043 (12)*	
H3A	0.850 (8)	0.182 (5)	0.103 (6)	0.12 (3)*	
H1B	0.210 (5)	0.695 (3)	0.427 (4)	0.043 (12)*	
H2B	0.051 (6)	0.440 (3)	0.771 (4)	0.049 (13)*	
H3B	0.836 (7)	0.274 (5)	−0.009 (4)	0.069 (17)*	

### Atomic displacement parameters (Å^2^)

**Table:**

	*U*^11^	*U*^22^	*U*^33^	*U*^12^	*U*^13^	*U*^23^
Ni1	0.0260 (4)	0.0160 (3)	0.0190 (3)	0.0037 (2)	−0.0020 (2)	−0.0023 (2)
C1	0.0293 (18)	0.0210 (17)	0.0308 (18)	0.0002 (13)	−0.0018 (15)	−0.0049 (13)
C2	0.0247 (18)	0.031 (2)	0.040 (2)	0.0061 (14)	−0.0065 (15)	−0.0046 (15)
C3	0.0285 (18)	0.0260 (18)	0.0344 (19)	0.0079 (14)	0.0048 (15)	−0.0010 (14)
C4	0.0249 (16)	0.0183 (16)	0.0203 (15)	0.0011 (12)	0.0068 (13)	0.0007 (12)
C5	0.0241 (17)	0.0199 (16)	0.0223 (16)	0.0026 (12)	−0.0016 (13)	−0.0007 (12)
C6	0.0260 (16)	0.0171 (15)	0.0211 (15)	0.0023 (12)	0.0062 (13)	0.0001 (12)
C7	0.0277 (17)	0.0209 (16)	0.0197 (16)	0.0004 (12)	0.0054 (13)	0.0008 (12)
C8	0.0285 (18)	0.0289 (18)	0.0218 (16)	−0.0021 (14)	0.0057 (14)	0.0005 (13)
C9	0.038 (2)	0.032 (2)	0.036 (2)	0.0010 (16)	−0.0030 (17)	−0.0087 (16)
C10	0.049 (3)	0.051 (3)	0.035 (2)	−0.005 (2)	0.0004 (19)	−0.0144 (18)
C11	0.031 (2)	0.052 (3)	0.039 (2)	−0.0002 (17)	−0.0001 (17)	0.0073 (18)
C12	0.033 (2)	0.038 (2)	0.034 (2)	0.0034 (16)	0.0022 (16)	−0.0022 (16)
N1	0.0311 (15)	0.0184 (14)	0.0242 (14)	0.0033 (11)	0.0031 (12)	−0.0016 (11)
N2	0.0275 (15)	0.0193 (14)	0.0239 (14)	0.0022 (11)	0.0030 (12)	−0.0017 (11)
N3	0.0363 (16)	0.0175 (14)	0.0217 (14)	0.0035 (11)	0.0015 (12)	−0.0013 (10)
N4	0.0356 (16)	0.0224 (15)	0.0203 (14)	0.0040 (12)	−0.0009 (12)	−0.0031 (11)
N5	0.0390 (19)	0.054 (2)	0.0289 (17)	−0.0109 (16)	−0.0033 (14)	0.0008 (15)
O1	0.0298 (13)	0.0263 (13)	0.0320 (14)	0.0000 (10)	−0.0057 (11)	0.0068 (11)
O2	0.0554 (17)	0.0220 (14)	0.0216 (12)	−0.0001 (12)	−0.0001 (12)	0.0005 (10)
O3	0.0514 (18)	0.0363 (16)	0.0345 (15)	0.0052 (13)	0.0128 (14)	−0.0034 (12)

### Geometric parameters (Å, °)

**Table:**

Ni1—O1^i^	2.063 (3)	C7—N2	1.347 (4)
Ni1—O1	2.063 (3)	C7—C8	1.456 (5)
Ni1—O2	2.083 (2)	C8—C12	1.389 (5)
Ni1—O2^i^	2.083 (2)	C8—C9	1.398 (5)
Ni1—N1	2.094 (3)	C9—C10	1.373 (6)
Ni1—N1^i^	2.094 (3)	C9—H9A	0.9300
C1—N1	1.330 (4)	C10—N5	1.331 (6)
C1—C2	1.379 (5)	C10—H10A	0.9300
C1—H1	0.9300	C11—N5	1.333 (5)
C2—C3	1.381 (5)	C11—C12	1.373 (5)
C2—H2	0.9300	C11—H11A	0.9300
C3—C4	1.380 (5)	C12—H12A	0.9300
C3—H3	0.9300	N3—N4	1.376 (4)
C4—C5	1.385 (4)	O1—H1A	0.836 (19)
C4—C6	1.472 (4)	O1—H1B	0.830 (18)
C5—N1	1.345 (4)	O2—H2A	0.828 (18)
C5—H5A	0.9300	O2—H2B	0.836 (19)
C6—N3	1.327 (4)	O3—H3A	0.82 (5)
C6—N2	1.344 (4)	O3—H3B	0.81 (4)
C7—N4	1.337 (4)		
			
O1^i^—Ni1—O1	180.00 (15)	N4—C7—N2	113.1 (3)
O1^i^—Ni1—O2	88.62 (11)	N4—C7—C8	121.9 (3)
O1—Ni1—O2	91.38 (11)	N2—C7—C8	125.0 (3)
O1^i^—Ni1—O2^i^	91.38 (11)	C12—C8—C9	116.5 (3)
O1—Ni1—O2^i^	88.62 (11)	C12—C8—C7	121.6 (3)
O2—Ni1—O2^i^	180.0	C9—C8—C7	121.9 (3)
O1^i^—Ni1—N1	90.86 (11)	C10—C9—C8	119.4 (4)
O1—Ni1—N1	89.14 (11)	C10—C9—H9A	120.3
O2—Ni1—N1	87.82 (11)	C8—C9—H9A	120.3
O2^i^—Ni1—N1	92.18 (11)	N5—C10—C9	124.1 (4)
O1^i^—Ni1—N1^i^	89.14 (11)	N5—C10—H10A	118.0
O1—Ni1—N1^i^	90.86 (11)	C9—C10—H10A	118.0
O2—Ni1—N1^i^	92.18 (11)	N5—C11—C12	124.1 (4)
O2^i^—Ni1—N1^i^	87.82 (11)	N5—C11—H11A	117.9
N1—Ni1—N1^i^	180.000 (1)	C12—C11—H11A	117.9
N1—C1—C2	122.5 (3)	C11—C12—C8	119.5 (4)
N1—C1—H1	118.7	C11—C12—H12A	120.2
C2—C1—H1	118.7	C8—C12—H12A	120.2
C1—C2—C3	119.1 (3)	C1—N1—C5	118.1 (3)
C1—C2—H2	120.5	C1—N1—Ni1	122.7 (2)
C3—C2—H2	120.5	C5—N1—Ni1	118.9 (2)
C4—C3—C2	119.2 (3)	C6—N2—C7	101.9 (3)
C4—C3—H3	120.4	C6—N3—N4	105.3 (3)
C2—C3—H3	120.4	C7—N4—N3	105.8 (3)
C3—C4—C5	118.1 (3)	C10—N5—C11	116.3 (3)
C3—C4—C6	122.0 (3)	Ni1—O1—H1A	125 (3)
C5—C4—C6	119.9 (3)	Ni1—O1—H1B	119 (3)
N1—C5—C4	122.9 (3)	H1A—O1—H1B	107 (3)
N1—C5—H5A	118.5	Ni1—O2—H2A	138 (3)
C4—C5—H5A	118.5	Ni1—O2—H2B	114 (3)
N3—C6—N2	114.0 (3)	H2A—O2—H2B	107 (3)
N3—C6—C4	121.9 (3)	H3A—O3—H3B	111 (3)
N2—C6—C4	124.1 (3)		
			
N1—C1—C2—C3	1.6 (6)	C4—C5—N1—C1	−0.1 (5)
C1—C2—C3—C4	−0.3 (5)	C4—C5—N1—Ni1	174.5 (2)
C2—C3—C4—C5	−1.0 (5)	O1^i^—Ni1—N1—C1	−129.6 (3)
C2—C3—C4—C6	179.1 (3)	O1—Ni1—N1—C1	50.4 (3)
C3—C4—C5—N1	1.3 (5)	O2—Ni1—N1—C1	−41.0 (3)
C6—C4—C5—N1	−178.8 (3)	O2^i^—Ni1—N1—C1	139.0 (3)
C3—C4—C6—N3	11.2 (5)	O1^i^—Ni1—N1—C5	56.0 (2)
C5—C4—C6—N3	−168.7 (3)	O1—Ni1—N1—C5	−124.0 (2)
C3—C4—C6—N2	−169.8 (3)	O2—Ni1—N1—C5	144.6 (2)
C5—C4—C6—N2	10.3 (5)	O2^i^—Ni1—N1—C5	−35.4 (2)
N4—C7—C8—C12	172.1 (3)	N3—C6—N2—C7	−0.4 (4)
N2—C7—C8—C12	−7.2 (5)	C4—C6—N2—C7	−179.4 (3)
N4—C7—C8—C9	−8.3 (5)	N4—C7—N2—C6	0.5 (4)
N2—C7—C8—C9	172.4 (3)	C8—C7—N2—C6	179.8 (3)
C12—C8—C9—C10	−0.7 (6)	N2—C6—N3—N4	0.1 (4)
C7—C8—C9—C10	179.7 (4)	C4—C6—N3—N4	179.2 (3)
C8—C9—C10—N5	−1.7 (7)	N2—C7—N4—N3	−0.5 (4)
N5—C11—C12—C8	−2.7 (6)	C8—C7—N4—N3	−179.8 (3)
C9—C8—C12—C11	2.8 (5)	C6—N3—N4—C7	0.2 (3)
C7—C8—C12—C11	−177.6 (3)	C9—C10—N5—C11	2.0 (6)
C2—C1—N1—C5	−1.3 (5)	C12—C11—N5—C10	0.2 (6)
C2—C1—N1—Ni1	−175.7 (3)		

Symmetry codes: (i) −*x*, −*y*+1, −*z*+1.

### Hydrogen-bond geometry (Å, °)

**Table:**

*D*—H···*A*	*D*—H	H···*A*	*D*···*A*	*D*—H···*A*
O1—H1A···N5^ii^	0.83 (2)	1.92 (3)	2.751 (4)	179 (3)
O1—H1B···N3^iii^	0.83 (3)	1.95 (3)	2.750 (4)	162 (3)
O2—H2A···O3^iv^	0.83 (3)	1.93 (3)	2.751 (4)	171 (3)
O2—H2B···N4^v^	0.84 (3)	1.96 (3)	2.791 (4)	169 (3)
O3—H3A···N2^vi^	0.82 (5)	2.10 (5)	2.911 (4)	170 (3)
O3—H3B···N4^vii^	0.82 (4)	2.20 (5)	2.944 (4)	151 (3)

Symmetry codes: (ii) *x*+1, *y*+1, *z*+1; (iii) *x*, *y*+1, *z*; (iv) −*x*+1, −*y*+1, −*z*+1; (v) −*x*, −*y*, −*z*+1; (vi) *x*+1, *y*, *z*; (vii) −*x*+1, −*y*, −*z*.
